# Motivations for Starting and Stopping PrEP: Experiences of Adolescent Girls and Young Women in the HPTN 082 Trial

**DOI:** 10.1007/s10461-025-04703-0

**Published:** 2025-06-03

**Authors:** Lisa Mills, Makhosazane Nomhle Ndimande-Khoza, Jennifer Velloza, Millicent Atujuna, Miria Chitukuta, Sybil Hosek, Hlukelo Chauke, Petina Musara, Nomvuyo Mangxilana, Prisca Mutero, Lerato Michelle Makhale, Thelma Tauya, Connie Celum, Sinead Delany-Moretlwe, the HPTN 082 study team

**Affiliations:** 1 Wits RHI, Faculty of Health Sciences, University of the Witwatersrand, 22 Esselen Street, Johannesburg, 2000 Republic of South Africa; 2 Department of Epidemiology and Biostatistics, University of California San Francisco, San Francisco, CA USA; 3 Desmond Tutu HIV Centre, Institute of Infectious Disease and Molecular Medicine, Faculty of Health Scienced, University of Cape Town, Cape Town, South Africa; 4 University of Zimbabwe Clinical Trials Research Centre, Harare, Zimbabwe; 5 Department of Medicine, Center for Dissemination and Implementation Science, University of Illinois College of Medicine, Chicago, IL USA; 6 Departments of Global Health, Medicine, Epidemiology, University of Washington, Seattle, WA USA

**Keywords:** HIV, Pre-exposure Prophylaxis, Africa, Women, Adherence, Social Factors

## Abstract

Oral PrEP effectiveness depends on consistent use during periods of potential HIV exposure, but adolescent girls and young women (AGYW) find this challenging. Data on PrEP use decision-making and alignment with risk among AGYW are limited. From 2016 to 2018, we conducted in-depth interviews with participants in HPTN 082, an open-label PrEP study in South Africa and Zimbabwe, to explore reasons for PrEP starts, stops, and restarts. Of 60 PrEP acceptors, 12 delayed acceptance, 15 used PrEP intermittently, 18 paused and restarted PrEP, and 13 permanently discontinued PrEP during 12-month follow-up. Perceived HIV vulnerability motivated PrEP start, but there was little evidence that fluctuating risk perception motivated prevention-effective use. PrEP stops were motivated by stigma, misconceptions and side effects; PrEP restarts were prompted by support from family, peers and clinic staff. Decision-making was related to social, gendered and normative influences, highlighting opportunities for psycho-educational support and multimedia campaigns to normalise HIV prevention.

## Introduction

Adolescent girls and young women (AGYW) in sub-Saharan Africa are disproportionately affected by HIV, and are 2.4 times more likely to acquire HIV than their male peers [1]. Oral pre-exposure prophylaxis (PrEP) is highly efficacious in preventing HIV infection, but consistent daily use and use during periods of HIV risk can be a challenge for AGYW [2–5]. High PrEP uptake has been observed among AGYW in recent PrEP demonstration projects, but discontinuation rates are also high within the first few months [6]. Open-label PrEP studies have looked at factors associated with PrEP use during follow-up, and have identified several barriers to consistent daily oral PrEP uptake and use including pill size, daily regimen, side effects, stigma,misconceptions, depressive symptoms, non-disclosure, lack of social support and access to clinics [7–16]. The influence of prevention-effective use and motivations related specifically to PrEP stops and restarts have been less explored.

Given the challenges that AGYW experience in adhering to PrEP, achieving prevention-effective adherence (periodic, effective use of PrEP during periods of potential HIV exposure) has been suggested as the important goal of an oral PrEP support strategy, and avoids conceptions of PrEP as a life-long treatment like antiretroviral therapy (ART) [17, 18]. Unlike ART, if used correctly during periods of exposure or vulnerability, oral PrEP can be paused and restarted as needed; however, prevention-effective adherence depends on AGYW having accurate knowledge of PrEP’s efficacy and ability to anticipate and align PrEP use with sexual behavioural patterns.

More work is needed across contexts and populations to understand AGYW motivations for and decision-making around PrEP at various points in the PrEP use journey. This may provide insight into the type of information and support AGYW need to make informed decisions about stopping and restarting oral PrEP, could prompt recommendations for use of longer-acting (LA) products and help understand potential patterns of use for LA products, especially among adolescents. Using data from HPTN 082, we explored AGYW’s motivations for starting, stopping and restarting PrEP.

## Methods

### Study Design

Qualitative interviews were conducted with a subset of participants enrolled in HPTN (HIV Prevention Trials Network) 082, a randomised open-label study assessing acceptance and adherence to daily oral PrEP [2]. Between 2016 and 2018, the trial enrolled 426 AGYW from Cape Town and Johannesburg, South Africa, and Harare, Zimbabwe. Women were eligible if they were aged 16–25 years, HIV negative, literate in one of the study languages, sexually active in the month prior to screening, and at risk for HIV as determined by an empiric HIV risk score, and PrEP-eligible [19]. Participants could enrol irrespective of their intention to start PrEP. Participants who did not start PrEP immediately were offered PrEP at each visit. Participants who accepted PrEP were randomised in a 1:1 ratio to either standard adherence support, or standard adherence support plus drug-level feedback counselling. Standard adherence support included adherence counselling at study visits, weekly short message service (SMS) reminders, provision of education materials and monthly in-person adherence support clubs. Those that received drug-level feedback had intracellular tenofovir diphosphate concentrations measured at week four and eight using dried blood spots; adherence counselling was provided on the basis of these drug concentrations at their next visit. Plasma tenofovir levels were tested at week 4, 8, 13, 26 and 52. A subset of 60 participants were recruited to participate in serial in-depth interviews regarding their PrEP use experiences.

## Study Setting and Context

HPTN 082 participants were recruited from high-density, low-income settings. In Cape Town and Harare, participants were recruited from communities on the outskirts of the city. In Johannesburg, participants were recruited from the densely populated inner-city communities. The HPTN 082 trial was initiated ahead of national guidelines recommending PrEP use for HIV prevention among AGYW in South Africa and Zimbabwe in December 2016 and PrEP was not widely available outside of the study [20, 21].

## Qualitative Sampling and Data Collection

Participants from each site were purposely sampled to include adherers, those that had difficulty with adherence, PrEP decliners and unique cases. Adherence was based on plasma drug level at week four, defining those that adhered well to PrEP (tenofovir > 40 ng/mL) and those that had difficulty adhering (tenofovir < 40 ng/mL). Unique cases were intentionally sampled and included participants from each site who reported social harm or were placed on product hold for protocol-specified safety reasons like pregnancy. Of those participants that were selected and interviewed, this analysis includes only those that initiated PrEP during the trial period. Selected participants were invited to take part in two serial individual in-depth interviews at the 13- and 26-week study visits. The purpose of conducting serial interviews was to track PrEP experiences over time. Between March 2017 and October 2018, female qualitative researchers conducted in-depth interviews using a standard interview guide in one of the preferred study languages (English, isiXhosa, isiZulu, Sesotho or Shona). Interviews were approximately 45 minutes in duration and topics covered motivations for taking or refusing PrEP, facilitators and barriers to PrEP adherence, PrEP use disclosure and the influence of social relationships on PrEP use, and community knowledge about PrEP. Interviews were audio-recorded, transcribed, translated into English, and checked by site teams for accuracy.

### Data Analysis

**Fig. 1:**
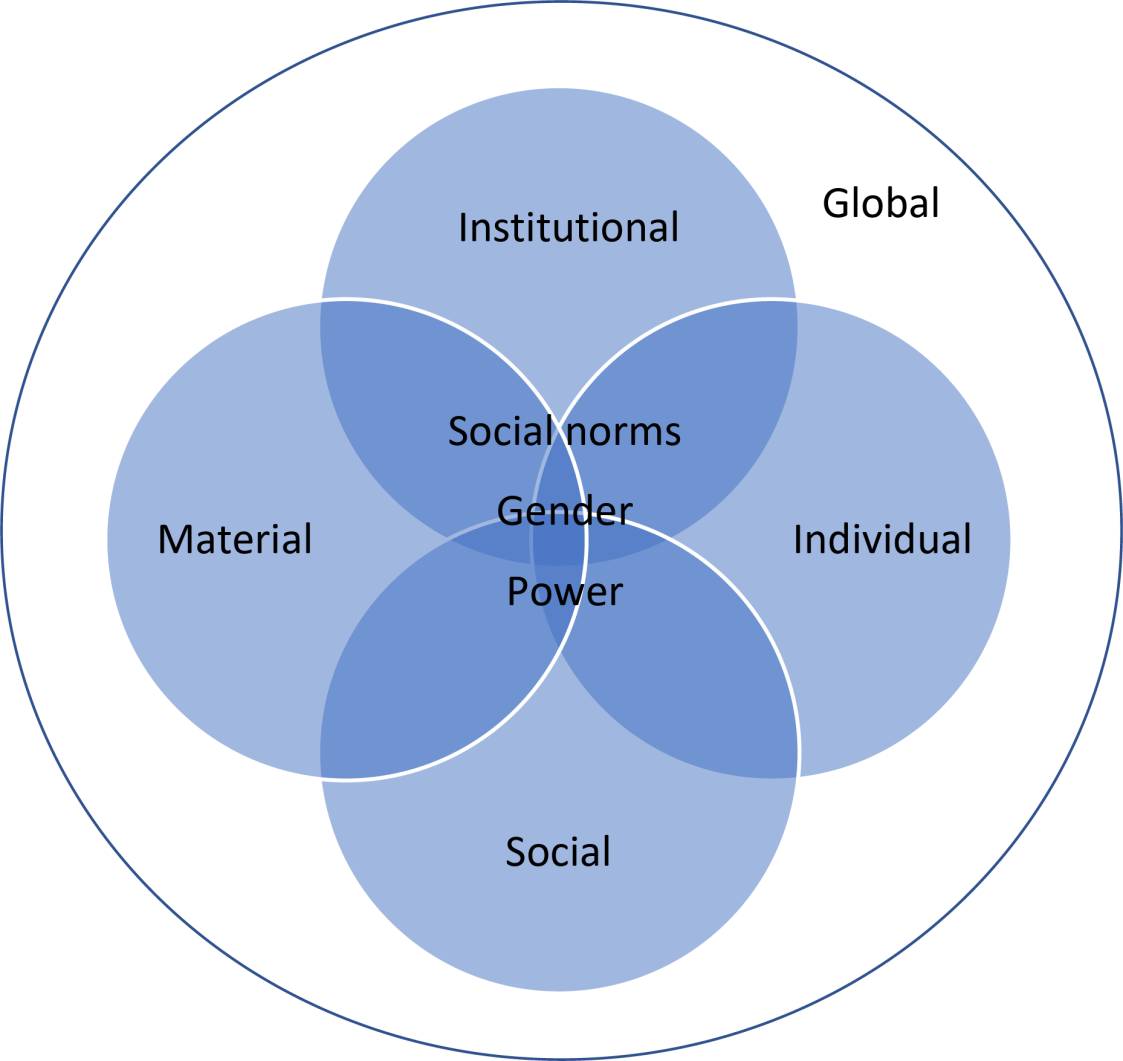
‘Dynamic framework for social change’. (Adapted from Cislaghi and Heise [24])

Transcripts were coded in NVivo (versions 11 and 12; QSR International, Melbourne, Australia) using a thematic, inductive approach [22]. A codebook was developed using open coding [23]. Each transcript was coded independently by one of eight qualitative researchers (JV, MNK, FS, MC, PM (Petina Musara), NM, LN, MA) each of whom had experience in qualitative coding. Coding was reviewed on approximately 20% of the transcripts to check alignment with the developed codebook. Disagreements were resolved during ‘reconciliation discussions’ with individual coders, until consensus was reached [22]. Codes relevant to this analysis were extracted and included motivations to take PrEP, motivations to delay and discontinue PrEP and self-reported adherence. Memos were written for each participant, detailing their reasons for starting, delaying, or stopping PrEP. Full transcripts were consulted again if motivations were unclear and to ensure experiences and timelines related to stopping and restarting were fully captured. Heise’s dynamic framework (Fig. 1), an adaptation of Bronfenbrenner’s ecological framework, was used to organise multi-level influences on AGYW’s PrEP use [24]. The framework identifies various possible domains of influence on health behaviour– individual (e.g., belief, skills), social (e.g., relationships, community), institutional (e.g., governance, legal system), material (e.g., access to assets, transport), and global (e.g., consumerism, technological innovation). These factors are understood as having interactional effects on behaviour, with the implication that behavioural change requires intervention across multiple domains. The dynamic adaptation of Bronfenbrenner’s ecological framework identifies an additional overarching embeddedness or influence of gender, power and social norms across all intersections and domains [24]. Participant quotations were extracted for representative themes. Pseudonyms were randomly assigned to participants.

The study was reviewed and approved by the research ethics committees at the Universities of the Witwatersrand, Zimbabwe, and Cape Town. All participants provided written informed consent in their preferred language, and at the Johannesburg site, participants younger than 18 provided assent with consent from a parent or legal guardian.

## Results

We interviewed 60 participants that initiated PrEP during the study period (Harare, *n* = 22, Johannesburg, *n* = 19, Cape Town *n* = 19). Twelve participants (20%) delayed PrEP initiation for some time after enrolment (“delayed acceptors”), 15 (25%) practiced intentional intermittent use (for example, intentionally taking PrEP once a week or every other day; excluding missed doses due to forgetting), 18 (30%) paused PrEP for a fixed period with the intention of discontinuing and then restarted, and 13 (22%) permanently discontinued PrEP during the trial. Some participants (*n* = 13) were in more than one of these categories during the 12-month follow-up period. Table 1 indicates the sociodemographic profile of AGYW who participated in the interviews, stratified by those whoself-reported accepting and continuing PrEP (reported no PrEP stops), and those who reported accepting PrEP but discontinued, paused or practiced intermittent PrEP use (reported PrEP stops). Most AGYW lived with their parents. There was a higher proportion of AGYW who reported PrEP stops among participants from Johannesburg and Harare compared to the Cape Town site.

**Table 1:** Socio-demographic characteristics of HPTN 082 participants in the qualitative study by self-reported PrEP use

Characteristics	Reported no PrEP stops (*n* = 24)	Reported intermittent PrEP use, pause and/or discontinuation (*n* = 36)	Total (*n* = 60)
Median age in years (IQR)	21 (20–22)	22 (20–24)	22 (20–23)
Harare	7 (32%)	15 (68%)	22 (100%)
Cape Town	9 (47%)	10 (53%)	19 (100%)
Johannesburg	7 (37%)	12 (63%)	19 (100%)
*Education*			
Completed primary school	6 (35%)	11 (65%)	17 (100%)
Completed secondary school	17 (40%)	26 (60%)	43 (100%)
*Occupation*			
Employed	3 (27%)	8 (73%)	11 (100%)
Unemployed	8 (35%)	15 (65%)	23 (100%)
Student	12 (46%)	14 (54%)	26 (100%)
*Relationship status*			
Single, no partner	2 (33%)	4 (67%)	6 (100%)
Married/living with a primary partner	3 (43%)	4 (57%)	7 (100%)
Dating, no primary partner	1 (25%)	3 (75%)	4 (100%)
Dating, primary partner	17 (40%)	26 (60%)	43 (100%)
*Living with*			
With parents	14 (44%)	19 (56%)	33 (100%)
With partner	4 (33%)	8 (67%)	12 (100%)
Alone	2 (50%)	2 (50%)	4 (100%)
With other^a^	3 (27%)	8 (73%)	11 (100%)

^a^With other refers to living with other family, children only, with housemates or other

The results have been organized according to Heise’s dynamic framework; however, individual, social, material, and institutional factors often intersect and are not always easily separated into distinct categories. Young women also usually provided multiple reasons for interruptions in PrEP use. Table 2 illustrates factors that influenced AGYW PrEP use behaviour, categorizing them across the domains of Heise’s dynamic framework and identifying intersections with underlying norms.

**Table 2:** Using the dynamic framework to diagnose factors that influence PrEP use behaviour (Adapted from Cislaghi and Heise [24])

Domains	Factor	Contribution to PrEP use	Embedded norms, and intersection with social and material influence
Individual	Perception and changes in perceived HIV vulnerability (partners’ risk, multiple partners, condom use, transactionalrelationships, vulnerability of young women to HIV and sexual violence)	Starting PrEP (less frequently motivated PrEP pause, restart and discontinuation)	• Men as ‘untrustworthy,’ attribution of individual risk to male partners • Limited power of young women to negotiate condom use • Expectations of condomless sex in transactional relationships • Association of condom use with infidelity • Normalization and expectation of sexual violence against women
	Fear of side effects	Delayed acceptance	• Myths/misconceptions about PrEP’s side effects i.e., PrEP causes weight gain, change in body shape, infertility, organ failure, increased vulnerability to HIV • Gendered standards of women as slim, shapely, fertile
	Experience of side effects	Intermittent use PrEP pause PrEP discontinuation	• Social pressure from family/belief that experiencing side effects indicates drug intolerance • Prioritization of school or employment over risk of side effects and poor performance
	Challenge/dislike of daily dosing	Intermittent use PrEP pause PrEP discontinuation	• Normalization of medicine as treatment for ‘sick’ people and not for healthy people
Social	Exposure to HIV/AIDS, (loosing parents or relatives to HIV/AIDS, exposure to orphanhood and poverty, experience of a friend seroconverting)	Starting PrEP	• ‘Old HIV/AIDS narratives’– association of HIV/AIDS with death, orphanhood and poverty
	Internalized or experienced stigma	PrEP pause	• Association of PrEP with ARVs i.e., use of ARVs as an indication of HIV infection • Use of PrEP or HIV prevention as an indication of ‘promiscuity’ or infidelity • Gendered practices and beliefs granting decision-making power to husbands, who may hold stigmatized notions of PrEP
	Intersection of travel and stigma, fear of unintended disclosure while travelling	Intermittent use	
	Partner or husband disapproval	PrEP pause PrEP discontinuation	
	Peer influence and encouragement, including reassurance about side effects	Starting and restarting PrEP	• Framing of PrEP daily dosing as ‘just like’ oral contraceptive/normalization of HIV prevention as part of routine sexual and reproductive healthcare
	Family and community support	Restarting PrEP	
Institutional	Clinic service and support (retention events, drug-level feedback) and support from clinic staff (encouragement, information sharing leading to increased understanding of risk)	Starting and restarting PrEP	

### Motivations for Starting PrEP

At the individual level, perceived HIV vulnerability was the predominant motivator for starting PrEP. A few Zimbabwean participants involved in transactional sex attributed their HIV vulnerability to having multiple partners. Among these participants, none reported a delay in accepting PrEP. Perceived HIV vulnerability was sometimes linked to concerns about condom use, including the experience or fear of condom breakage, inconsistent use (especially when drinking alcohol), and difficulty negotiating condom use. While individual-level behaviour did influence beliefs about vulnerability, HIV vulnerability was mostly spoken about with reference to partner vulnerability, indicating an influence of normative expectations and social-level perceptions about personal HIV vulnerability. Participants described having an ‘untrustworthy’ partner, experiencing infidelity, contracting a sexually transmitted infection (STI), not knowing a partners’ HIV status, or spending time away from a partner. One participant explained, “I have been dating one guy, and I think he may give me problems. So let me join [the study]. The way I view it, it’s for my own safety.” (Setso, 24, Johannesburg).

Individual-level factors related to geographic location and demographics sometimes contributed to perceived HIV vulnerability. One participant illustrated the intersecting impact of individual, material and normative factors on PrEP start: “We as young women, as for our age group, we are the ones who are found to have the disease [HIV/AIDS] because we will be having so much pressure. Maybe you have to take care of your family, so you will be saying there is nothing that I can do, let me just sleep with that person fair [without protection] because that person will be taking care of you.” (Zendaya, 24, Harare).

Social-level factors could also motivate PrEP initiation. Participants often described being introduced to PrEP by peers who were already enrolled in the study. Some participants knew individuals living with HIV or had experienced the passing of parents or family from HIV/AIDS and were motivated to avoid the same experience for themselves or their children. A few South African participants also expressed interest in PrEP due to high levels of sexual violence in their neighbourhoods.

## Delaying PrEP Initiation

Delays to PrEP initiation were predominantly due to individual beliefs or preferences relating to fear of side effects and/or a dislike for daily pill-taking. There was no data indicating that delays were caused by low perceived HIV vulnerability due to absence of sexual activity. Social-level influence was the central motivation in participants decisions to initiate PrEP after delaying. A few participants said they had waited to see if their peers experienced side effects before starting. Encouragement from family or gaining permission was another reason for delayed initiation, “My father wasn’t there; he was in Bloemfontein [South African city]. So, I couldn’t, like I had to wait for him to come back and then explain to them what I joined and explain to him and see if he will agree or not, so he agreed and said that there’s no problem if it will help me” (Mahlaku, 20, Cape Town). Clinic staff were also identified as sources of information and reassurance, and helped participants with increased understanding of PrEP’s efficacy, “I realised that I needed to be told more about PrEP, instead of listening to my friend. When they [clinic staff] explained PrEP to me, I understood what they were talking about. The best thing was for me to also take PrEP to be protected.” (Sphe, 20, Johannesburg). After initiating PrEP, the majority of delayed acceptors reported no stops in their PrEP use. Conversely, many of those who accepted PrEP at enrolment reported later intermittent use, pauses and/or discontinuation of PrEP.

## Motivations for Deliberate Intermittent Use of PrEP

Fifteen participants reported intentionally taking PrEP intermittently, or skipping doses on a regular basis, for example taking PrEP once a week, every other day or once every few days (excluding missed doses due to forgetfulness). There was little data to suggest that intermittent use was motivated by changes in sexual activity; only one intermittent user suggested that they were more motivated to take their pill when they were sexually active, “Another thing is that for me when I want to have sex, like I usually say that I want those pills.” (Berenice, 20, Cape Town).

Intermittent use of PrEP was most frequently attributed to individual-level experiences of side effects (gastrointestinal upset, fatigue, headaches) and/or a dislike for pill-taking. Aversion to pills was attributed to the large size of the pill or experiencing daily dosing as boring or irritating, “So like this pill is bitter and it bores me. I usually cut it in half and take one side. And then another half I would flush it; I usually did that.” (Berenice, 20, Cape Town). Stigma was a social factor for intentional intermittent use, mostoften related to anticipated stigma and fear of inadvertent disclosure when travelling. Participants reported intentionally leaving PrEP at home or did not take doses when travelling. Embarrassment and fear were often linked to the noisy PrEP pill bottle which drew attention. Participants said they wanted to avoid explaining what PrEP is used for and feared that others would mistake their PrEP for ARVs, “There will be many people. So, for you take a pill you can’t first explain that the pills do this and this. Ah it’s difficult to start to explain to people. So, the bottle embarrassed me.” (Danai, 24, Harare).

In most cases, intermittent use of PrEP was reported as a consistent pattern of use. Women reported a few occasions where their intermittent use was limited to the period following PrEP initiation, and this was largely attributed to side effects. These participants often reported more regular dosing once side effects had subsided. A few participants reported switching to regular dosing due to support from the clinic, including information-sharing sessions, drug-level feedback, retention events, and encouragement from staff or peers. One intermittent user was reassured by the counsellor about a rumour that stopping PrEP can cause an ‘incurable disease’: “I didn’t take it [PrEP] that well last month and then I went there [spoke to a counsellor] and then I was informed [that there is no incurable disease].” (Vusi, 19, Cape Town).

### Motivations for Pausing or Discontinuing PrEP

Eighteen participants reported pausing PrEP (stopping PrEP for a fixed period with the intention of discontinuing, but later deciding to restart) and thirteen participants reported discontinuing PrEP (stopped PrEP and had not restarted at the time of the last interview). Four participants paused PrEP for a protocol specified indication (abnormal lab value or pregnancy), and two safely resumed. A further two paused because they were unable to attend clinic visits, and two participants discontinued due to seroconversion.

Sexual inactivity was sometimes cited as a motivation to pause or discontinue PrEP, and in these instances, women often cited intersecting social influence such as partner or family disapproval, “I feel that it [PrEP] made him feel that I don’t trust him, yeah. So, I… this caused a bit of a tension. Interviewer: So that has affected your motivation? Participant: Yeah, and the fact that I am not really sexually active, yeah, so, I found it very unnecessary for me to take the pill when there is nothing you know, actually going on.” (Amogelang, 24, Johannesburg).

Pausing and discontinuation was sometimes attributed to individual-level preferences related to drug attributes including side effects or dislike for taking pills, “That pill is distasteful, so I told myself this pill is not nice, and it makes me hungry. So, I told myself that I should discontinue using it, and I told myself that I’m not sick to take pills every day, so I decided that I should stop taking it.” (Celine, 20, Cape Town).

Social-level and normative influences were frequently cited as a motivation for temporary PrEP pauses. Stigmatised beliefs and misconceptions included rumours that participants were living with HIV and on ARVs, that using HIV prevention indicates ‘promiscuous’ behaviour, PrEP can give you HIV, and PrEP causes a change in body shape (e.g., large stomach, hunchback, large upper body, bad structure), infertility or organ damage. Partner disapproval resulted in temporary and permanent PrEP stops. PrEP use was said to cause a partner to question the participant’s fidelity, or to believe that the participant doubted their own fidelity. Two married Zimbabwean participants had discontinued PrEP at the time of the final interview, attributing discontinuation to their husband’s disapproval. The respective spouses had expressed that PrEP is used by ‘promiscuous’ individuals or sex workers: “He just asked me what I have to do with the pills. Then he started to say that maybe I will be ‘prostituting’ in Zimbabwe. Then I just stopped taking the pills.” (Zendaya, 24, Harare).

Women often reported multiple motivations for pausing or discontinuing PrEP. All participants that attributed PrEP discontinuation to side effects also revealed additional social, normative or material influences. Chenai (24, Harare) reported pressure from her aunt to discontinue due to challenges with side effects, “I would vomit and spend the whole day nauseous, and I wouldn’t want to eat. My aunt was also complaining too much saying, ‘If you are always vomiting what does it mean? Don’t keep on forcing things that aren’t working for your body.’” Another participant revealed material influences and concerns about work performance, stating that she had chosen not to restart after she became employed and wanted to avoid feeling fatigued at work.

### Motivations for Restarting PrEP

A few participants resumed PrEP due to resuming sexual activity and reported taking appropriate measures to ensure prevention-effective PrEP use (testing with their partner and/or taking PrEP for the recommended period before resuming sexual activity). Low perceived vulnerability and a desire to avoid initial PrEP side effects could prevent PrEP restart, “It was because I was single at that time and also because I was thinking about this thing of the side effects, again starting from scratch.” (Dikeledi, 21, Johannesburg).

Social and institutional support from clinic staff, peer participants and family was frequently cited as a motivation to restart PrEP. This was the case for those who paused PrEP due to drug attributes such as side effects and dislike for pill-taking, and those who paused due to social or normative factors related to stigma or disapproval. One participant that stopped PrEP due to community rumours that she was taking ARV’s was encouraged to return to the study clinic by her mother who stated that “Go, because the man will bring you the disease when you are what? Seated at home?” (Sophie, 23, Harare). Support from clinic staff included clarifying misconceptions about PrEP. A participant who paused PrEP due to an unusually heavy menstrual period, later restarted after clinic staff explained that heavy menstruation was a result of her contraceptive method.

Support from family could motivate PrEP restart in cases of partner disapproval, “At first, he [participant’s partner] didn’t appreciate it because he didn’t understand. I then took my friend, and we explained to him. Because my family, including my grandmother and grandfather know that we are taking PrEP, they sat down with him and told him, that is when he accepted it.” (Nyasha, 20, Harare). Support from family did not always prompt PrEP restart. Two Zimbabwean participants that permanently discontinued PrEP due to husband disapproval, explained that their parents were supportive of PrEP use, but ultimately advised them to obey their husbands.

## Discussion

Perceived HIV vulnerability was an important motivator for PrEP initiation, but relative to normative, social or drug attributes (pill aversion, experience/perception of side effects), was not a common motivator for PrEP stops and restarts. Decisions for delaying, pausing or discontinuing PrEP were predominantly influenced by desires to conform to social norms, with social support from clinic staff and social networks playing a motivating role for starting or restarting PrEP.

Some HPTN 082 participants reported practicing safe prevention-effective adherence, indicating that AGYW understand HIV vulnerability and make appropriate choices, and confirming the primary findings of HPTN 082 (association between any HIV risk and high PrEP adherence) [6, 25]. Relative to motivations based on drug characteristics or social factors, however, there was comparatively little reference to stopping and restarting PrEP due to thoughtful prevention-effective use based on sexual activity. Similarly among Kenyan AGYW, HIV risk trajectories were not associated with PrEP adherence trajectories, and in the HPTN 082 sample, only a subset were able to align PrEP use with risk [25, 26]. These findings indicate that AGYW could benefit from prevention-effective counselling adherence. AGYW should be provided guidance on starting and stopping PrEP safely, with encouragement and guidance on retesting and restarting PrEP if or when there is restart in sexual intimacy.

The majority of AGYW’s reported decisions to start PrEP were influenced by their perceived vulnerability to HIV, which they attributed to ‘untrustworthy’ partners. Women often expressed normative expectations of male infidelity in intimate relationships [27], which compelled young women in the sample to take responsibility for HIV prevention. Among South African participants, the influence or fear of sexual violence on their decision to use HIV prevention also reflects high rates of sexual violence in the country and a disturbing social norm about violence against women [28, 29]. The interviews confirmed that routine conflation of condom use with infidelity and expectations of condomless sex in transactional relationships impacted perceived HIV vulnerability [12, 30, 31]. Norms in transactional relationships may also limit women’s ability to decline sex altogether [32] which couldcontribute to increased HIV vulnerability for women involved in transactional relationships.

A less frequent but important motivation for starting PrEP was losing family members, becoming orphaned as a result of AIDS, or having a friend acquire HIV. Fear-based motivations can be attributed to decades of ‘cycling’ between HIV/AIDS, premature death, and poverty [33, 34]. While these narratives can motivate HIV prevention uptake, stigmatising associations of HIV with illness and poverty are detrimental to early detection and ART adherence [34]. More positive narratives are needed to de-stigmatise HIV treatment and prevention and normalise it as part of routine, comprehensive sexual and reproductive healthcare (SRH). With improved HIV treatment outcomes, fear-based motivations may naturally decline. Although we did not find related evidence in our data, reduction of perceived severity of HIV infection may contribute to decreased prevention behaviours [35–37]. This emphasizes that PrEP should be part of comprehensive SRH services which promote a combination prevention approach including use of condoms, regular HIV testing and STI screening. Positive, empowering narratives that highlight the role of PrEP in increasing decision-making power and promoting self-care and overall sexual and reproductive wellness may also help to foster more sustained PrEP engagement among AGYW [38].

Beliefs and misconceptions were frequently embedded among AGYW’s fear or experience of side effects and subsequent decisions to delay, pause or stop PrEP. Misconceptions about PrEP’s side effects should be understood in context of broader social mistrust of new pharmaceutical interventions [7, 39, 40], ambivalence about clinical trials and research [7, 41] and association of daily drug use with illness [7, 39, 41, 42]. The impact of archetypal expectations of the ‘feminine’ (i.e., to be slim, shapely and fertile) [43, 44] on AGYW’s decision-making and health-seeking behaviour was also evident among women who stopped PrEP due to believing it can cause infertility, weight gain, and changes in body shape. Fears about side effects are well documented barriers to PrEP use in open-label extension studies [7, 9, 12, 16]. Encouragingly, all participants in the HPTN 082 sample who delayed or paused PrEP due to side effects, did eventually start or restarted PrEP following encouragement from family, peers or clinic staff.

Challenges with drug attributes (i.e., aversion to pill-taking and perceived/experienced side effects) have been frequently cited as impacting PrEP adherence, but only resulted in permanent PrEP discontinuation for a few participants in HPTN 082, all of whom also expressed intersecting motivations related to social or material factors [7, 9, 16, 39, 41]. Intermittent use or temporary pauses related to side effects or pill taking were usually limited to the period following PrEP initiation. Similarly, among Ugandan AGYW, most PrEP stops as a result of side effects were not permanent [12].

Stigmatised associations of PrEP use with infidelity, ‘promiscuity’ or having HIV caused many AGYW to temporarily stop PrEP. Challenges to PrEP adherence have frequently been attributed to stigma [7, 9, 12, 14, 16, 39, 41]. Intermittent PrEP use as a result of anticipated stigma was especially pertinent to AGYW travelling away from home. This may be exacerbated or impacted by less PrEP knowledge in rural areas [45]. With the support of friends, family or clinic staff, most AGYW overcame challenges related to stigma. Stigmatising ‘promiscuity’ narratives within spousal relationships may warrant additional investigation and support, as this can result in permanent discontinuation, as was reported by two Zimbabwean participants. Partner disapproval or lack of support has been linked to unequal relationship power and the potential infringement of female HIV prevention use on relationship power and status quo [45, 46].

Social support from family members was an important motivator for acceptance after initial delays or restarting PrEP. Strong social networks, disclosure, and the ability to navigate social scrutiny have similarly been identified as facilitators of PrEP use and restart, while lack of social support or stability is a commonly identified barrier [13, 14, 16, 45–49]. Lack of social support has been linked to family or partners having insufficient information and comprehension of product purpose and safety, but may improve over time following information-sharing with social support figures [14, 46, 50]. In a separate qualitative analysis on PrEP disclosure and adherence in the HPTN 082 sample, AGYW were better able to disclose PrEP use over time due to study-provided support including education on disclosure skills [51]. The negative impact of spousal disapproval, and the positive potential of social support highlight the possible benefits of mass media, community and family-level interventions that normalize and destigmatize HIV prevention. Offering disclosure support or involving partners or guardians in counselling sessions may also be useful in enhancing the supportive potential of social networks. Parental consent, engagement, and support for product use contributed to high retention and product use among AGYW in an 18-month multi-site cross over trial of the dapivirine ring and daily oral PrEP [52]. This strategy, however, should be considered alongside the legal and ethical complexities surrounding adolescent PrEP access. In South Africa, adolescents aged 12 and older can legally acquire PrEP without parental approval if assessed as having sufficient maturity, while in Zimbabwe, lack of clear guidelines means that adolescents might require parental consent up to the age of majority at 18 years [53, 54]. Alternative strategies may be needed where parental consent is a barrier for younger adolescents [55]. This could include community engagement campaigns that target stigma among elders, adopting proxy consent models, policy and legal advocacy, and improving legal literacy among healthcare providers.

Information sharing and support from clinic staff, and opportunities to engage with other AGYW PrEP users at the clinic were important facilitators for restarting PrEP after pausing. Other studies have similarly highlighted the benefit of adherence counselling in overcoming PrEP use challenges, and suggest that lack of clinic contact could lead to drops in adherence, especially between quarterly refill periods [11, 12, 45]. Patient support programs that allow online or face-to-face engagement with other PrEP users, or with clinic staff could be beneficial to African AGYW. More evidence is needed on counselling approaches like drug-level feedback, adherence counselling including addressing fears about side effects, cognitive behavioural therapy and tailored approaches specific to context, age and relationship status [56]. The use of digital information-sharing tools may complement and enhance the effect of responsive clinical care. A client-facing HIV prevention decision support tool (DST) has been piloted and has shown initial success in HIV prevention uptake and persistence among African AGYW [57]. More evidence is needed on the efficacy and implementation possibilities for interactive and digital media educational tools.

The potential of discreetly accessing long-acting injectable HIV prevention modalities during clinic visits could resolve PrEP use challenges or interruptions due to daily dosing fatigue, disapproval and stigma, andintermittent use due to fear of inadvertent disclosure while travelling. Interventions that enhance social support and incorporate information-sharing at the clinic level may remain relevant, however, as the uptake of long-acting HIV prevention methods may still be susceptible to hesitancy as a result of HIV prevention stigma, mistrust of new pharmacological interventions, and misconceptions or fears about side effects.

### Strengths and Limitations

The results capture a diversity of motivations and challenges in PrEP use, offering insight to important questions regarding low adherence to oral PrEP among African AGYW. The results relied on self-reported data, which introduces the possibility of recall and social desirability bias. Participants also received support and participated in study activities not available to young women who obtain PrEP in standard public delivery settings. Potential bias is countered by the large sample size and longitudinal data captured in two interviews for each participant. It would not be possible to coincide drug-level feedback collection points with self-reported periods of non-use for comparison, as exact dates and lengths of self-reported PrEP delays, stops and restarts were not recorded during in-depth interviews. The purpose of this qualitative research is to understand young women’s priorities and challenges during decision making about PrEP use, and not to corroborate self-reported data with quantitative data.

## Conclusion

Analysis of African AGYW’s PrEP starts, stops and restarts has highlighted that while prevention-effective adherence is used by a few women, it is not the primary reason for periodic oral PrEP use, and decision-making is inextricably tied to social and normative influences. AGYW would benefit from support, education and skills to understand and cope with stigma, fear of unintended disclosure, gender norms, spousal disapproval, and myths about side effects. There may also be value in interventions that can support young women to better operationalise prevention-effective use so that they can start and stop safely. Online decision support tools and interactive digital platforms may be useful and could also potentially address the need for more regular clinical contact and connection with peer PrEP users. In addition to individual-level behavioural change interventions, investigation and exploration of support interventions that address broader contextual determinants of PrEP use is needed. Interventions targeted at individuals should be coupled with mass media and community-level interventions that normalise HIV treatment and prevention and address misconceptions and suspicion of research and pharmacological intervention.

## Data Availability

All relevant data are within the manuscript. The protocol is available at https://www.hptn.org/sites/default/files/2016-05/HPTN082_FINAL_Protocol_12082015_0.pdf. All deidentified transcripts of interviews are stored at Wits RHI, South Africa. They are available from the study corresponding author on reasonable request.

## Code availability (software application or custom code)

NVivo versions 11 and 12; QSR International, Melbourne, Australia.
